# Alternative Splicing at a NAGNAG Acceptor Site as a Novel Phenotype Modifier

**DOI:** 10.1371/journal.pgen.1001153

**Published:** 2010-10-07

**Authors:** Alexandre Hinzpeter, Abdel Aissat, Elvira Sondo, Catherine Costa, Nicole Arous, Christine Gameiro, Natacha Martin, Agathe Tarze, Laurence Weiss, Alix de Becdelièvre, Bruno Costes, Michel Goossens, Luis J. Galietta, Emmanuelle Girodon, Pascale Fanen

**Affiliations:** 1 INSERM, Unité U955, Créteil, France; 2 Université Paris-Est, Faculté de Médecine, UMR-S 955, Créteil, France; 3 Laboratorio di Genetica Molecolare, Istituto G. Gaslini, Genova, Italy; 4 AP-HP, Groupe Henri-Mondor Albert-Chenevier, Service de Biochimie-Génétique, Créteil, France; 5 Service de Pédiatrie I, Hôpitaux Universitaires de Strasbourg, Hôpital de Hautepierre, Strasbourg, France; The Hospital for Sick Children and University of Toronto, Canada

## Abstract

Approximately 30% of alleles causing genetic disorders generate premature termination codons (PTCs), which are usually associated with severe phenotypes. However, bypassing the deleterious stop codon can lead to a mild disease outcome. Splicing at NAGNAG tandem splice sites has been reported to result in insertion or deletion (indel) of three nucleotides. We identified such a mechanism as the origin of the mild to asymptomatic phenotype observed in cystic fibrosis patients homozygous for the E831X mutation (2623G>T) in the *CFTR* gene. Analyses performed on nasal epithelial cell mRNA detected three distinct isoforms, a considerably more complex situation than expected for a single nucleotide substitution. Structure-function studies and *in silico* analyses provided the first experimental evidence of an indel of a stop codon by alternative splicing at a NAGNAG acceptor site. In addition to contributing to proteome plasticity, alternative splicing at a NAGNAG tandem site can thus remove a disease-causing UAG stop codon. This molecular study reveals a naturally occurring mechanism where the effect of either modifier genes or epigenetic factors could be suspected. This finding is of importance for genetic counseling as well as for deciding appropriate therapeutic strategies.

## Author Summary

Mild disease outcomes associated with premature termination codons can result from at least three different mechanisms, but none of these mechanisms explain the mild phenotype observed in some patients. Subtle differences in alternative transcripts have recently been reported at NAGNAG tandem acceptor motifs, which can be detected in 30% of human genes. We provide the first experimental evidence of premature termination codon removal by alternative splicing at a NAGNAG acceptor splice site. Our study emphasizes the biological significance of such alternative splicing in the context of disease-causing mutations and defines a new phenotype-modifying mechanism that buffers nonsense mutations.

## Introduction

Premature termination codons (PTCs) are usually associated with severe phenotypes. However, mild disease outcomes can occur by at least three different mechanisms [1]. First, translation can be initiated at an internal start codon located downstream from the PTC [2]. Second, PTCs can trigger nonsense-mediated mRNA decay (NMD), a pathway that protects the cell from aberrant transcripts [3]. Third, nonsense-associated alternative splicing (NAS) [4] can remove the exon harboring the PTC.

Subtle changes in alternative splicing events have recently been reported at particular tandem acceptor splice sites, NAGNAG sites (where N represents any nucleotide) [5]–[7]. The use of the intron proximal or distal splice site results in the production of two distinct isoforms distinguished by three nucleotides (NAG). This alternative splicing could result in the creation or deletion of a stop codon [5]. The latter event would thus constitute another mechanism of PTC removal, but has never been described in human pathophysiology.

Splice events at short-distance tandem sites are widespread and contribute to transcriptome and proteome complexity [5]. NAGNAG acceptor motifs are present in 30% of human genes and several studies based on computational analysis using expressed sequence tag (EST) databases showed that at least 5% of human genes contain an experimentally confirmed NAGNAG tandem site [5]. A subtle splice event associated with Stargardt's disease 1, in which a mutation in the *ABCA4* gene produced an indel of one amino acid in 50% of the transcripts from one patient, has been described [8]. Nonetheless, the involvement of mutations that alter tandem sites have not been extensively studied in disease [9]. Therefore, to explore further the effect of mutations located within a NAGNAG acceptor motif, we studied the cystic fibrosis transmembrane conductance regulator gene (*CFTR*). Querying the Tandem Splice Site DataBase (TassDB), a comprehensive online database dedicated to recognizing tandem acceptor sites, identified two NAGNAG motifs in *CFTR*. Mutations in the *CFTR* gene, which encodes a cAMP-regulated Cl^−^ channel located at the apical membrane of epithelial cells, cause cystic fibrosis (CF). CF is the most common severe autosomal recessive genetic disorder in Caucasians [10] and affects the physiology of the lung, gastrointestinal tract, reproductive organs, and sweat glands. Mutations in the *CFTR* gene induce a continuum of phenotypes ranging from mild manifestations with isolated features such as congenital bilateral absence of the vas deferens (CBAVD), or nasal polyposis, to severe disease symptoms. Therefore, CF is a good model to identify novel modifier mechanisms [11]. Here, we show that alternative splicing at a tandem acceptor site removes a premature UAG stop codon and leads to synthesis of a functional protein. This novel PTC removal mechanism explains the mild phenotype detected in several patients.

## Results/Discussion

### Family pedigree

**Figure 1:**
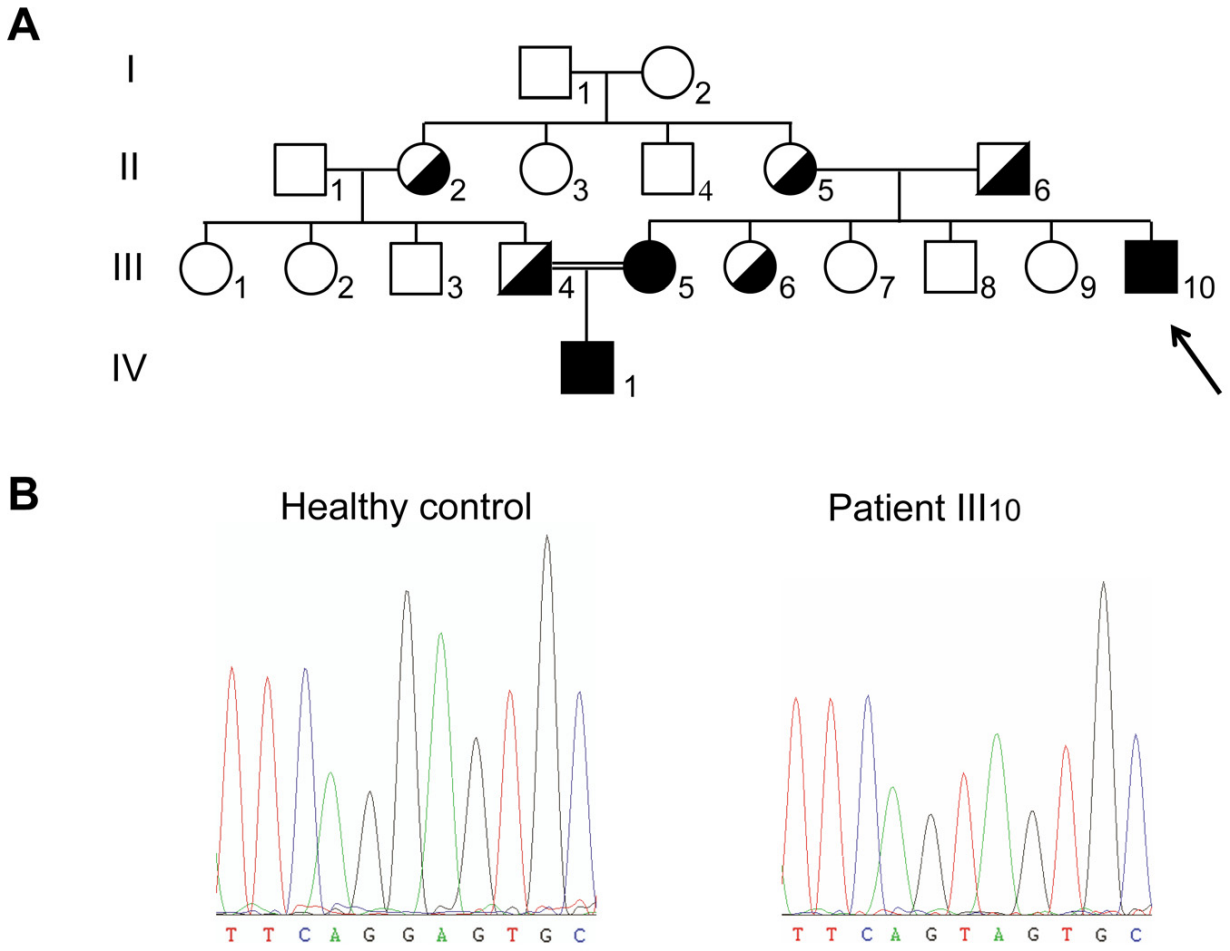
Pedigree and genotype of the patients. (A) Family pedigree. Identified carriers are indicated in half blackened symbols and CF patients in blackened symbols. Consanguinity between individuals III_4_ and III_5_ is highlighted with a double line. (B) Sequence analysis performed on the genomic DNA from a healthy control and patient III_10_.

In this study, we focused on the rare E831X mutation (2623G>T) which affects the first nucleotide of exon 14a which forms part of one of the two NAGNAG acceptor sites detected in the *CFTR* gene. We had the opportunity to study a consanguineous family of Turkish origin which included three patients (Figure 1A) homozygous for the E831X mutation (Figure 1B). To evaluate the functional consequences of this mutation in a tandem splice motif, we performed transcript analysis on epithelial cells obtained from patient III_10_ (Figure 1A) by nasal brushing, which is the least invasive technique and provides the most reliable cells to study *CFTR* mRNA.

### Direct transcript analysis

**Figure 2:**
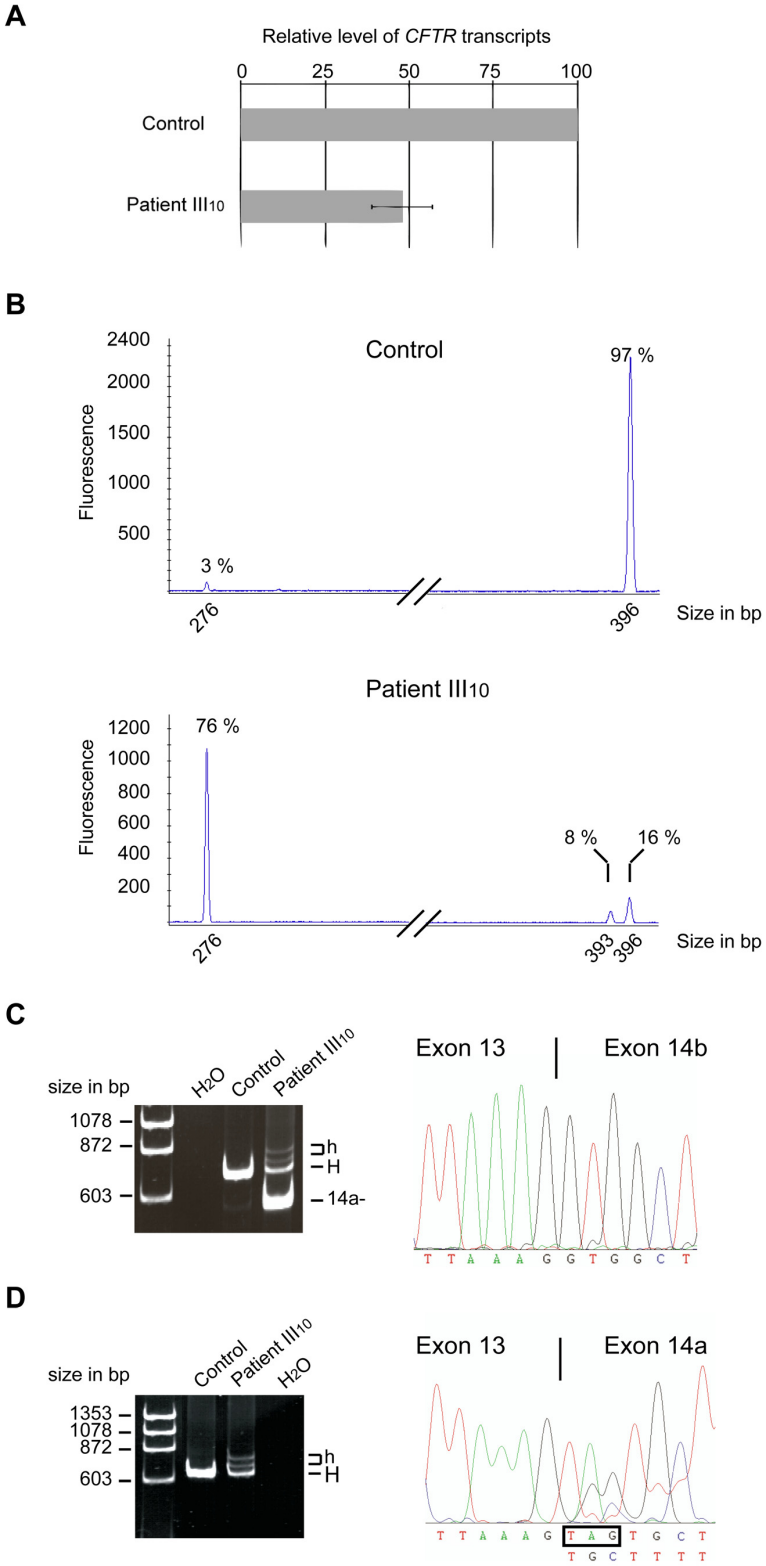
Direct transcript analysis. (A) qRT-PCR analysis performed on total RNA obtained from nasal epithelial cells of patient III_10_ or three healthy controls. Levels were normalized to the amount of *KRT18* and compared to the normalized level of the control individuals. Data represent the mean±SE of at least two independent measurements performed in triplicate. (B) An example of capillary electrophoresis analysis of RT-PCR products obtained after 30 cycles from control samples (n = 3) or patient III_10_. The corresponding size and relative amount of each peak are indicated. The peak size corresponds, respectively, to exon 14a skipped mRNA (276 bp), to full-length mRNA (396 bp), and to the mRNA lacking the UAG stop codon (393 bp). (C) RT-PCR performed with a forward primer in exon 13 and reverse primer within exon 17 on RNA from patient III_10_ and a healthy donor. Sequences were obtained from the excised band denoted 14a-. “H” designates the homoduplex bands of both 14a+ isoforms and “h” the heteroduplex bands. (D) RT-PCR performed with a reverse primer within exon 14a and forward primer in exon 13 on RNA from patient III_10_ and a healthy donor. Sequences were obtained from the excised homoduplex band (“H”); “h” designates heteroduplex bands.

Mutations that generate PTCs can reduce the steady-state level of mRNA via nonsense-mediated decay (NMD) [3]. Total RNA from nasal epithelial cells of patient III_10_ was first quantified to evaluate the amount of *CFTR* transcripts. Quantitative analysis by real-time PCR was normalized to that of *keratin 18* (*KRT18*), a marker of ciliated and secretory epithelial cells [12], [13]. The results clearly showed that the level of *CFTR* mRNA was reduced by half in the sample from patient III_10_ compared to three control samples (48%±9% compared to WT, Figure 2A), showing that the *CFTR* mRNAs were subject to NMD.

To evaluate the mRNA pattern associated with the 2623G>T mutation, a semi-quantitative RT-PCR analysis was performed and the products were analyzed by capillary electrophoresis. In control samples, two peaks could be detected: a major peak corresponding to the full-length mRNA (97%±0.5%) and a minor peak corresponding to exon 14a skipping (3%±0.5%) (Figure 2B), a feature of *CFTR* splicing that has been described previously [14]. In the patient III_10_ sample, a more complex pattern appeared, with the presence of three distinct peaks (Figure 2B) that were subsequently identified by sequencing: a major peak corresponding to exon 14a skipping (76%±2%) (Figure 2B and 2C) and two additional peaks corresponding to a full-length mRNA containing the stop codon (16%±2%) and an mRNA lacking these three nucleotides (8%±0.8%) (Figure 2B and 2D).

Hence, the 2623G>T substitution induced two effects: a reduced level of full-length mRNA containing the stop codon by NMD and the generation of additional mRNA isoforms. These mRNAs were identified as an mRNA lacking exon 14a, a full-length mRNA containing the premature UAG stop codon, and a third mRNA lacking the three nucleotides encoding the UAG stop codon.

### Hybrid minigene splicing assays

**Figure 3:**
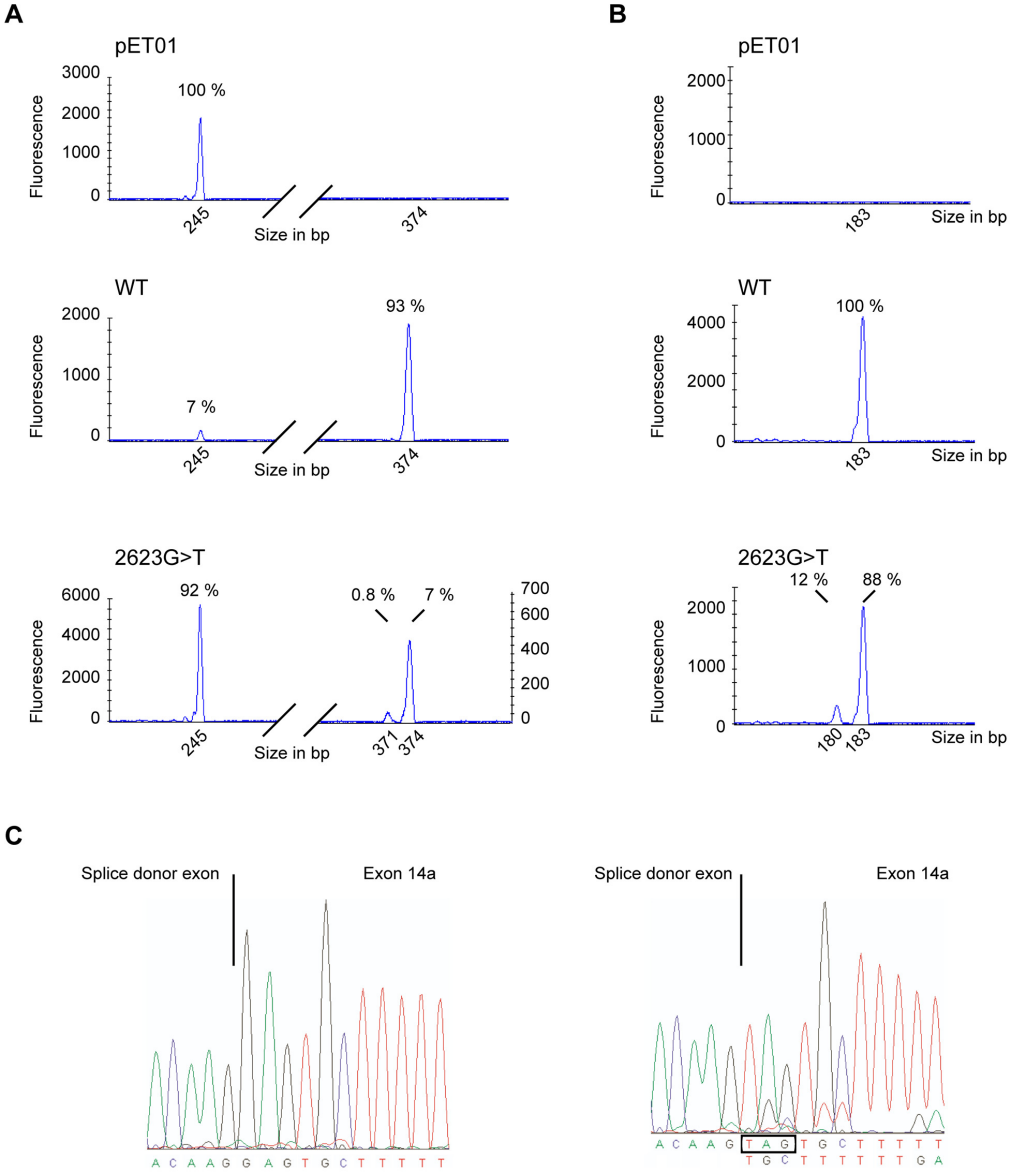
Hybrid minigene splicing assays. (A) Examples of capillary electrophoresis analysis of RT-PCR products obtained after 21 cycles. RNA was purified from BEAS-2B cells transfected with empty plasmid (pET01), minigene containing exon 14a (WT) or mutant exon 14a (2623G>T). RT-PCR was performed using a FAM-labeled forward primer located within the splice donor exon and a reverse primer within the splice acceptor exon of the pET01 plasmid. The corresponding size and relative amount of each peak is indicated. The peak at 245 bp corresponds to exon 14a skipped mRNA, at 374 bp to full-length mRNA and at 371 bp to mRNA lacking the UAG stop codon. (B) Examples of capillary electrophoresis analysis of RT-PCR products obtained with a FAM-labeled forward primer located within the splice donor exon of the pET01 plasmid and a reverse primer within exon 14a. The corresponding size and relative amount of each peak are indicated. The peak at 183 bp corresponds to full-length mRNA and at 180 bp to the mRNA lacking the UAG stop codon. (C) Sequences were obtained using unlabeled forward primer using the 21 cycle RT-PCR products from (B).

To investigate whether the alternative splicing was due to the 2623G>T mutation, we constructed a hybrid minigene containing *CFTR* exon 14a and approximately 400 nucleotides of its flanking intronic regions. After transfection in the bronchial epithelial cell line BEAS-2B, RT-PCR samples were separated by capillary electrophoresis analysis and isoforms were identified by sequencing. As in the direct transcript analysis, the wild-type construct revealed a low level of exon 14a skipping (7%±0.8%). The 2623G>T substitution increased exon 14a skipping up to 92%±2%. In addition, two peaks could be detected corresponding to full-length mRNA (7%±2%) and to the isoform lacking three nucleotides (0.8%.±0.5%) (Figure 3A and 3C). To focus on these two lower abundance isoforms, a reverse primer within exon 14a was used to amplify mRNAs containing exon 14a specifically. A single peak was detected in mRNA obtained from cells transfected with the wild-type construct, whereas two peaks were detected in mRNA from cells transfected with the 2623G>T construct. The major peak corresponded to full-length mRNA containing the UAG stop codon (88%±0.5%) and the mRNA in the minor peak lacked these three nucleotides (12%±0.5%) (Figure 3B and 3C). The ratio between these two isoforms was comparable using either reverse primer. The relative amounts of the three isoforms differed from the amounts measured for endogenous *CFTR*, but the minigene construct containing the mutated exon 14a sequence reproduced the *in vivo* splicing pattern. Therefore, the single nucleotide substitution (2623G>T) is the main determinant of alternative splicing at this site.

### Identification of a functional CFTR protein isoform

**Figure 4:**
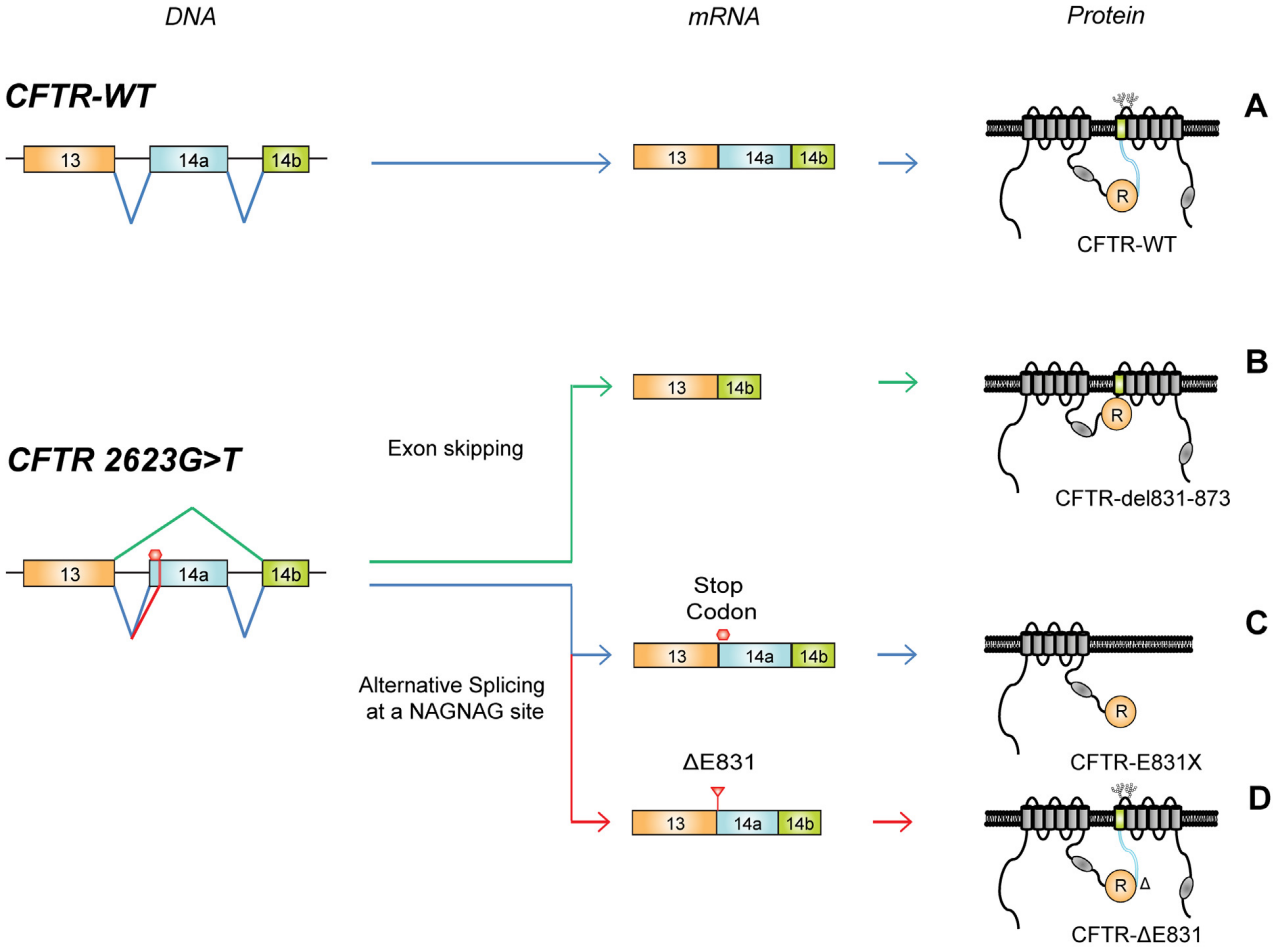
A single nucleotide substitution generating three distinct proteins. (A) CFTR-WT processing from DNA to protein. The R domain is encoded by exon 13 (orange), whereas exon 14a encodes the region linking the R domain to the seventh transmembrane segment (blue). The two glycosylation sites are indicated between the seventh and eighth transmembrane segment. The 2623G>T mutation located at the first nucleotide of exon 14a induces skipping of exon 14a producing CFTR-del831-873 (B). Alternative splicing at the NAGNAG site generates mRNA including the stop codon encoding truncated CFTR-E831X (C), and mRNA lacking the stop codon leading to CFTR-ΔE831 (D).

Recognition of the 3′ splice site upstream of exon 14a generates full-length mRNA leading to synthesis of the entire CFTR protein. The CFTR channel is composed of two transmembrane spanning domains, two nucleotide binding folds, and a regulatory domain (R domain). The regulatory domain is encoded by exon 13 (orange) and exon 14a encodes the region linking the regulatory domain to the seventh transmembrane segment (blue) (Figure 4A). The 2623G>T mutation which occurs at the first base of exon 14a induced multiple splicing defects, including exon skipping. An mRNA lacking exon 14a encodes a protein missing the linker domain between the R domain and the seventh transmembrane segment (CFTR-del831-873, Figure 4B). In addition to the full-length mRNA containing the UAG stop codon, alternative splicing at the NAGNAG site generated an mRNA lacking this stop codon. The resulting proteins would be either truncated after the regulatory domain (CFTR-E831X, Figure 4C) or missing one amino acid (CFTR-ΔE831, Figure 4D), respectively.

**Figure 5:**
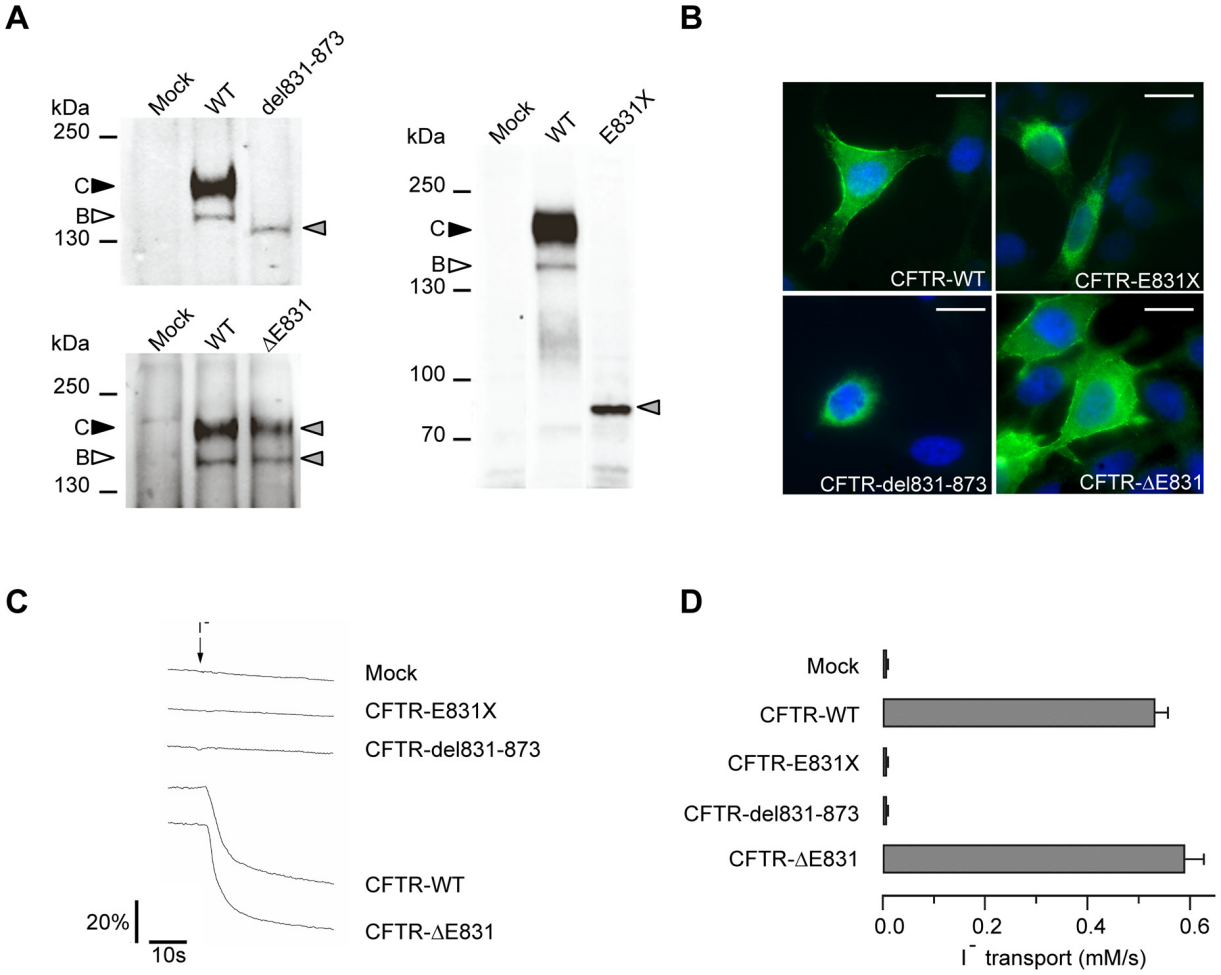
Characterization of the three CFTR mutant proteins. (A) Western blot analysis of HEK293 cells transiently transfected with CFTR-WT, CFTR-del831-873 (upper left panel), or CFTR-ΔE831 (lower left panel). Western blot analysis of HEK293 cells stably expressing CFTR-WT or CFTR-E831X (right panel). Filled and empty arrowheads indicate the fully-glycosylated (170 kDa) and core-glycosylated (140 kDa) CFTR, respectively. Grey arrowheads indicate mutant proteins. (B) Immunostaining performed on HeLa cells transiently transfected with the indicated constructs. CFTR was stained with MM13-4 anti-CFTR and an AlexaFluor 488 conjugated secondary antibody (green), nuclei were visualized using DAPI (blue). Scale bars: 10 µm. (C) Representative cell fluorescence recordings from HEK293 cells transiently expressing the halide-sensitive YFP (scale bar reports the percentage of total cell fluorescence). Extracellular addition of I^−^ (arrow) caused YFP quenching with a rate proportional to the rate of I^−^ influx and CFTR activity. (D) Summary of data obtained from the functional assay reporting rates of I^−^ transport for the indicated constructs in HEK293 cells. The bars report the transport of iodide as determined from the maximal rate of cell fluorescence decrease (mean±SE, n = 8–12).

The processing of CFTR can be assessed by examining its glycosylation state [15]. Western blot analysis of wild-type CFTR protein, transiently (Figure 5A, left panels) or stably (Figure 5A, right panel) expressed in HEK293 cells, revealed two bands. The diffuse band of 170 kDa (band C) corresponds to the mature, fully glycosylated protein and the thin band of approximately 140 kDa (band B) represents the core-glycosylated immature CFTR. Similar analysis of transiently expressed CFTR-del831-873 revealed a unique thin band indicating a maturation defect (Figure 5A, higher left panel) that was further investigated using an N-Glycosidase F assay (Figure S1). After enzymatic treatment of CFTR-WT, both bands B and C were converted to the lower apparent molecular weight non-glycosylated band A. CFTR-del831-873 was not affected by the enzymatic treatment, indicating an absence of glycosylation of this protein, thus identifying the first naturally occurring non-glycosylated CFTR mutant.

Stably expressed CFTR-E831X protein was detected as a single band at the expected size (Figure 5A, right panel), while transiently expressed CFTR-ΔE831 showed a normal maturation profile with the presence of both bands B and C (Figure 5A, lower left panel). Immunostaining of transiently transfected HeLa cells confirmed a processing defect in both the CFTR-del831-873 and CFTR-E831X mutant proteins as they could only be detected in intracellular compartments close to the nucleus, whereas both CFTR-WT and CFTR-ΔE831 showed clear cell surface staining (Figure 5B). Finally, functional assays showed the absence of CFTR-dependent anion transport in cells expressing either CFTR-del831-873 or CFTR-E831X. In contrast, a high level of anion transport was observed with CFTR-WT and CFTR-ΔE831, in accordance with the biochemical and immunocytochemical results above (Figure 5C and 5D). Therefore, we conclude that CFTR-ΔE831 represents the functional form accounting for the mild phenotype observed within this family.

### Phenotypes associated with the E831X mutation

CF was suspected in the first year of life in patient III_10_ (Figure 1A) due to recurrent bronchitis, and this was confirmed by a positive sweat test (70 mmol/L). The patient was tested and found to be homozygous for the nonsense mutation E831X. After familial study, CF was diagnosed in his 18-year-old sister (III_5_), who also had a positive sweat test and the same genotype. However, the two siblings, now aged 13 and 30, are in good health with no evidence of pancreatic insufficiency, and have normal lung function tests. The female patient married her first cousin who was found to be an E831X carrier. Their son (IV_1_) is homozygous for the E831X mutation. Now 5 years of age, he presents with a positive sweat test (74 mmol/L), but yearly clinical assessments are normal (Table 1).

**Table 1:** Phenotypes associated with E831X mutations.

Age at diagnosis, gender: M, F	Ethnic origin	Genotype	Sweat test* (mmol/l)	Phenotype	References
Birth, F	-	E831X/G551D	100	Early childhood: MI, PI, and lung infectionsAdulthood: PI and no pulmonary symptoms	15
Twins 13y, M	Turkish	E831X/591del18	89/94	Recurrent nasal polyps	16
Adult, M	Turkish	E831X/D110H	-	CBAVD	17
Adult, M	Turkish	E831X/1677delTA	-	CBAVD	17
Adult, M	Turkish	E831X/ΔF508	92/94	CBAVDPS and mild lung disease	18
Adult, M	Portuguese	E831X/ΔF508	-	CBAVD	19
First year, F	-	E831X/ΔF508	100	PS and no lung involvement	French registry
First year, M	Turkish	E831X/E831X	70	PS and mild lung disease	This study III_10_
Adult, F	Turkish	E831X/E831X	70	PS and no lung involvement	This study III_5_
Neonatal diagnosis, M	Turkish	E831X/E831X	74	PS and mild lung disease	This study IV_1_

Abbreviations: CBAVD, congenital bilateral absence of the vas deferens; MI, meconium ileus; PI, pancreatic insufficiency; PS, pancreatic sufficiency.

*Sweat test: normal values <30 mmol/l and border line values 30–60 mmol/l.

Interestingly, this mutation was first described in a female CF patient carrying the severe missense substitution G551D on the other allele. E831X was considered as a severe mutation because she presented with meconium ileus at birth, a neonate pulmonary infection and an elevated sweat test [16]. Given our results, we requested yearly clinical assessments, which indicated no pulmonary exacerbation, an almost normal chest radiograph, moderate pancreatic insufficiency and normal abdominal ultrasound. Thus, her clinical outcome was better than expected at birth. This mutation was also reported in two 13-year-old male twins of Turkish origin carrying an in-frame deletion on the other allele (591del18) [17]. These twins had persistent nasal polyps and elevated sweat tests, but no pancreas or lung involvement [17]. Subsequently, E831X was reported to be the allele present in cohorts of men with CBAVD and mild to severe mutations, such as ΔF508, for the other allele [18]–[20]. These clinical observations and our data bring the deleterious nature of the E831X mutation into question; but how can the minor functional CFTR-ΔE831 isoform lead to such mild phenotypes? The minimal level of *CFTR* mRNA required to maintain normal function differs between organs, with the vas deferens being the most sensitive tissue [21]. CFTR-ΔE831 mRNAs did not appear to reach this threshold in all tissues, leading to mild phenotypes such as nasal polyposis or CBAVD in compound heterozygotes. In the nasal epithelial cells of patient III_10_, mRNAs encoding functional CFTR-ΔE831 were estimated at 8%. As total *CFTR* transcripts were reduced to 48% compared to WT levels, this amount can be corrected down to 4%, a level comparable to previous studies associated with mild lung disease [22], [23]. However, the relative level of each transcript may differ between patients, as the same PTC has been shown to elicit NMD with variable efficiency in CF nasal epithelial cells, thus reducing the amount of full-length mRNA containing the stop codon [13].

### Analysis of the splice site context in the case of a NAGNAG motif

**Figure 6:**
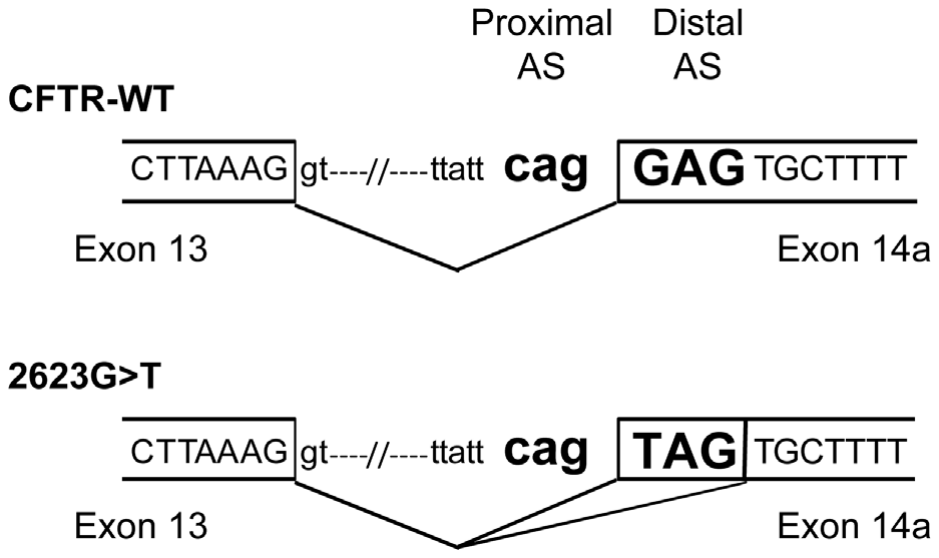
Schematic representation of the intron 13/exon 14a junction. Proximal and distal acceptor sites (AS) are indicated both on the WT and 2623G>T sequences. The tandem splice acceptor site is highlighted in bold. A broken line represents splicing events.

The intron 13/exon 14a boundary, illustrated in Figure 6, shows the presence of a proximal acceptor site in the intron and a distal acceptor site in the exon, typical of a NAGNAG motif. The use of the proximal or distal acceptor site is regulated by multiple factors. First, the strength of the acceptor site depends on the site itself (CAG>TAG>AAG), the polypyrimidine tract, and the branch point sequences. In addition, the branch point sequence-to-NAGNAG region in the 3′ tandem splice site was shown to participate in 3′ splice-site selection [24]. This selection could also be modulated by RNA-binding proteins because exonic or intronic splicing enhancer (ESE, ISE) and silencer (ESS, ISS) sites have been described as overabundant in the vicinity of tandem 3′ splice sites compared to constitutively spliced exons [25]. Lastly, the nucleotides composing the NAGNAG acceptor site are also tightly implicated in the recognition of either the proximal or distal splice site. NAGNAG acceptor sites have been classified with respect to their splicing plausibility. Plausible sites allow the use of both acceptor sites, whereas implausible ones allow the use of a single site [26]. The 2623G>T substitution converts the NAGNAG acceptor motif from an implausible CAGGAG (use of the proximal site) into a plausible CAGTAG motif (use of either proximal or distal splice sites). Therefore, the mutation would favor the use of the distal splice site, resulting in bypass of the PTC and explaining the mechanism leading to an mRNA lacking three nucleotides (Figure 6).

*In silico* analysis using software dedicated to analyzing NAGNAG motifs (BayNAGNAG) [27] predicted a shift in the probability of using both the proximal and distal acceptor sites from 0.2% to 81.2%, consistent with our *in vivo* results (Table 2). The first description of a disease-causing mutation in a NAGNAG splice site was reported in the *ABCA4* gene [8], before the description of the NAGNAG motif by Hiller *et al.* in 2004 [5]. This mutation (2588G>C) was shown to produce two isoforms leading to either an indel of Gly863 or to a missense defect, Gly863Ala, by shifting the NAGNAG motif from an implausible sequence (TAGGAG) into a plausible sequence (TAGCAG). Similarly, *in silico* analysis predicted an increase in the probability of using either distal or proximal acceptor sites from 0.1% to 57.7% (Table 2). Our study demonstrated that disease-causing mutations in NAGNAG are predictive of alternative splicing, as previously proposed for single nucleotide polymorphisms in NAGNAG acceptors [26]. Therefore, in the absence of mRNA samples, such splicing events could be anticipated by *in silico* analysis.

**Table 2:** *In silico* analysis using BayNAGNAG software.

	Probability of using proximal acceptor site		Probability of using distal acceptor site		Probability of using both acceptor sites	
	WT	MUT*	WT	MUT*	WT	MUT*
*CFTR* gene	99.8%	17.1%	0	1.7%	0.2%	81.2%
*ABCA4* gene	99.8%	39.9%	0	2.3%	0.1%	57.7%

*MUT = 2623G>T in the *CFTR* gene and 2588G>C in the *ABCA4* gene.

The removal of a deleterious PTC could apply to many genes, as the configuration we have described is frequent in the human genome. Indeed, NAGGAG represents the most frequent NAGNAG motif recognizable upstream of a coding exon [5]. Querying the TassDB retrieved 4882 occurrences of the NAGGAG motif with GAG in a coding exon, representing 4597 genes. Among these NAGGAG motifs, 52.2% (n = 2551) are in intron phase 0, a configuration that leads to the first amino acid of the exon being a Glu, as shown in this study. We can therefore hypothesize that bypass of a deleterious PTC could occur when similar GAG to TAG mutations affect one of the 2551 NAGGAG motifs within the human genome.

### Concluding remarks

In addition to contributing to proteome plasticity [9], alternative splicing at tandem 3′ acceptor sites can also result in *in vivo* removal of a premature UAG stop codon. This feature differentiates the UAG stop codon from UAA or UGA and may help explain phenotype/genotype discrepancies. The novel PTC removal mechanism would be expected to lead to mild phenotypes, with the deletion of a single amino acid being potentially less deleterious than a truncating mutation. This study also emphasizes the biological significance of alternative splicing at tandem acceptor sites in the context of disease-causing mutations. Subtle splicing events could be considered simply as noise tolerated by the cell [28]; however, considering point mutations at NAGNAG acceptor sites clearly provides evidence of their functional relevance. Alternative splicing at these acceptor sites could define a novel phenotype-modifying mechanism buffering deleterious nonsense mutations. Overall, this study highlights the importance of thoroughly characterizing the molecular defects in patients with milder than expected phenotypes. Indeed, such molecular studies can reveal naturally occurring mechanisms where modifier genes or epigenetic factors have been suspected to have an effect.

## Materials and Methods

### Ethics statement

Informed consent was obtained from all subjects and the local ethics committee approved the study.

### mRNA purification

Cells obtained from nasal brushings were immediately transferred into RNA-Later buffer (Qiagen) and total RNA was purified as recommended by the manufacturer using QiaQuick Spin columns (Qiagen). Two independent RT-PCR assays were performed with 400 ng of total RNA for each sample (Applied Biosystems).

### Quantitative real-time PCR assays

*CFTR* and *KRT18* primers and probes (TaqMan FAM/NFQ-MGB probe format) were designed by Applied Biosystems. PCR reactions contained the TaqMan Gene Expression Assay Mix, TaqMan Universal PCR Master Mix, no AmpErase UNG, and 1 µl cDNA (or 1 µl of DNase/RNase free water for the No Template Control) in a final volume of 20 µl. Samples were placed in 96-well plates and amplified in an ABI 7900HT Sequence Detection System (Applied Biosystems). Amplification conditions were 10 min at 95.0°C, followed by 40 cycles of 15 s at 95.0°C and 1 min at 60.0°C. All reactions were run in triplicate and each sample was run in two QPCR assays. To correct for variations in the amount of input RNA and efficiency of the reverse transcription, *KRT18* (which is specifically expressed in ciliated and secretory epithelial cells) was quantified and results were normalized to these values. Relative amounts of *CFTR* mRNA were measured using the 2 ^−ΔΔCT^ method [29]. A control sample was chosen as a calibrator, *i.e.*, as the baseline for the comparative results.

### Experimental validation and quantification of splice variants

Semi-quantitative RT-PCRs were performed using 1 µl cDNA templates with a sense FAM-labeled primer in exon 13 (5′-AGTGTCACTGGCCCCTCAG-3′) and a reverse primer in exon 17 (5′-GTGTCGGCTACTCCCACGTA-3′) or in exon 14a (5′-CATGTAGTCACTGCTGGTATGCT-3′) in a 20 µl total volume. PCR conditions were 94°C for 5 min followed by linear phase amplification of 29 cycles or 30 cycles at 94°C for 20 sec, 60°C for 20 sec, and 72°C for 20 sec. All samples were then extended at 72°C for 1 min and, finally, cooled to 4°C in a 9700 thermocycler (Applied Biosystems). Capillary electrophoresis analysis (GeneScan) was performed using 1 µl of the diluted PCR mixture (1/40) added to 18.5 µl of formamide and 0.1 µl of ROX 400 HD fluorescent size standards (Applied Biosystems). The mixture was then denatured at 95°C for 5 min and cooled to 4°C. Amplified products were separated on an ABI 3130 XL DNA analyzer using 3130 POP6 and analyzed with the GeneMapper 4.0 software (Applied Biosystems). Ratios of splicing isoforms were determined as the peak area for the *CFTR* isoform divided by the total peak areas for the three isoforms. Data represent the mean±SE of at least two independent measurements performed in triplicate.

### Sequencing of mRNA isoforms

For sequencing of the different splice variants, PCR was performed using 1 µl of cDNA templates with a sense primer in exon 13 (5′-AGTGTCACTGGCCCCTCAG-3′) and a reverse primer in exon 17 (5′-GTGTCGGCTACTCCCACGTA-3′) or in exon 14a (5′-CATGTAGTCACTGCTGGTATGCT-3′) using the same conditions as described previously for 40 cycles. Following RT-PCR, products were resolved on a 2% agarose gel and bands of interest were excised and purified using a gel extraction kit (Promega). Samples were subsequently sequenced using both forward and reverse primers.

### Cell culture and transfection

HEK293 and HeLa cells were grown at 37°C, 5% CO_2_ in DMEM medium supplemented with 10% SVF and 1% PS. BEAS-2B cells (CRL-9609) cells were grown in LHC-8 medium supplemented with 10% fetal calf serum and 1% Penicillin Streptomycin. Cells were transfected with Lipofectamine 2000 according to the manufacturer's instructions. HEK293 cells stably expressing CFTR-WT or CFTR-E831X were selected using Zeocin (50 µg/mL).

### Constructs and mutagenesis

CFTR-WT cDNA was subcloned in pTracer [30] and CFTR-E831X, CFTR-ΔE831 and CFTR-del-831-873 were generated by site-directed mutagenesis (Stratagene) and each construct was sequenced. A fragment comprising exon14a, 387 bp of the upstream intron 13 and 371 bp of the downstream intron 14a was PCR amplified from genomic DNA obtained from a healthy volunteer or from patient III_10_ using the following primers: 5′-GGACCCCTGAAGAAACAGGT-3′ and 5′-GCCTTCTACTTTGAGCTTTCG-3′. PCR products were subcloned into pCR II (Invitrogen) and inserts sequenced before subcloning into the pET01 vector (Mobitec) at the BamHI and XbaI restriction sites.

### Hybrid minigene splicing assays

Minigenes containing either the exon 14a-WT or the 2623G>T mutation and flanking introns (387 bp upstream and 371 bp downstream of exon 14a) were transfected in BEAS-2B cells seeded in 6-well plates. Total RNA was purified as recommended by the manufacturer using QiaQuick Spin columns (Qiagen). The mRNA concentrations were measured using a NanoDrop spectrophotometer. The mRNA (1.5 µg in a final volume of 20 µl) was DNAse treated for 30 min at 37°C before heat denaturation of the enzyme (Quiagen). Treated mRNA (400 ng) was used to perform RT-PCR using the High capacity cDNA Reverse Transcription kit (Applied Biosystems). Ten percent of the RT-PCR product was PCR amplified using primers specific to the splice donor and splice acceptor exons of the pET01 plasmid (5′-FAM-GTGACAGCTGCCAGGATCG-3′ and 5′-CAGTGCCAAGGTCTGAAGGT-3′). PCR was conducted at 94°C for 5 min followed by linear phase amplification of 20 cycles or 21 cycles at 94°C for 20 sec, 60°C for 20 sec, and 72°C for 20 sec. All samples were then extended at 72°C for 1 min and, finally, cooled to 4°C in a 9700 thermocycler (Applied Biosystems). Capillary electrophoresis analysis of the PCR products was performed as previously described. Ratios of splicing isoforms were determined as the peak area of the considered isoform divided by the total peak areas. Sequencing was performed on the 21 cycle PCR products generated with the internal primer using an unlabeled forward primer located within the splice donor exon of the pET01 plasmid. Data represent the mean±SE of at least two independent measurements performed in duplicate.

### IP and WB analysis

HEK293 cells were plated on 100 mm dishes and transiently transfected with CFTR-WT, CFTR-del831-873 or CFTR-ΔE831. Immunoprecipitation was performed as previously described [31] with a C-terminal directed anti-CFTR antibody (24-1, R&D systems). Cells stably expressing CFTR-E831X or CFTR-WT were lysed in 1X RIPA buffer and samples containing 30 µg total protein were analyzed by Western blot. After gel electrophoresis and transfer, membranes were probed using the MM13-4 antibody (Millipore) directed against the N-terminal region of CFTR. Direct recording of the chemiluminescence was performed using the CCD camera of the GeneGnome analyzer and quantification using the GeneTools software (Syngene BioImaging Systems, Synoptics Ltd).

### N-Glycosidase F treatment

HEK293 cells were plated on 60 mm dishes and transiently transfected with CFTR-WT or CFTR-del831-873. Cells were washed twice with ice cold PBS and lysed in N-Glycosidase F buffer containing 20 mM sodium phosphate, pH 7.5, 0.1% SDS, 50 mM β-mercaptoethanol, and 1% Igepal, supplemented with protease inhibitors. N-Glycosidase F (3 Units, Roche) was added to lysates and incubated overnight at 37°C and one tenth of the volume of each sample was analyzed by Western blot.

### Immunocytochemistry

HeLa cells were seeded on glass coverslips and transfected with the appropriate construct. The following day, cells were fixed using ice-cold methanol. Cells were permeabilized (1% BSA, 0.1% TritonX-100 in PBS) and, then, incubated with the primary antibody, MM13-4 anti-CFTR, diluted 1/200, for 2 h at room temperature. Secondary antibody (1/500 dilution, AlexaFluor 488 conjugated, Invitrogen) was then added and incubated 1 h. Coverslips were mounted using Vectashield mounting medium containing DAPI (4, 6-Diamino-2-phenylindol) and analyzed using a Leica DMR epifluorescence microscope.

### YFP-based functional assays

CFTR activity was determined in transiently transfected HEK293 cells using the halide-sensitive yellow fluorescent protein YFP-H148Q/I152L as described previously [32]. Cells were plated in 96-well microplates (2.5×10^4^ cells/well) and co-transfected with CFTR and halide-sensitive YFP. The CFTR functional assay was carried out 48 h after transfection. Cells were incubated for 30 minutes with PBS containing forskolin (20 µM) before being transferred to an Olympus IX 50 fluorescence microscope (Chroma; excitation: HQ500/20X; emission: HQ535/30M; dichroic: 515 nm), equipped with a photomultiplier tube (Hamamatsu) for detection of fluorescence. Cell fluorescence was continuously measured before and after addition of NaI (final NaI concentration: 100 mM). The signal was digitized using a PowerLab 2/25 acquisition system (ADInstruments). Cell fluorescence recordings were normalized to the initial average value measured before addition of NaI. Signal decay was fitted to a double exponential function to derive the maximal slope corresponding to initial influx of I^−^ into the cells. Maximal slopes were converted to rates of change in intracellular I^−^ concentration (in mM/s) using the equation: d[I^−^]/dt = K_I_[d(F/F_0_)/dt] where K_I_ is the affinity constant of YFP for I^−^ and F/F_0_ is the ratio of the cell fluorescence at a given time vs. initial fluorescence [33].

### *In silico* analysis

Bayesian network predictions of splicing outcomes at NAGNAG tandem acceptors in wild-type and mutant conditions were calculated using the BayNAGNAG software, (http://www.tassdb.info/baynagnag/form.html).

### TassDB queries

Query for the number of genes containing a NAGGAG motif:

SELECT count(distinct GeneID) FROM Gene G, Transcript T, SS2Transcript2SED SS2Tr2SED, SpliceSite SS, SpliceEventData SED WHERE G.ID = T.GeneID AND T.ID = SS2Tr2SED.TranscriptID AND SS2Tr2SED.SpliceSiteID = SS.ID AND SS2Tr2SED.SpliceEventDataID = SED.ID AND SS.Type = ‘acceptor’ AND SED.NumEESTs> = 0 AND SED.NumIESTs> = 0 AND ((SS.pattern = ‘CAGGAG’) OR (SS.pattern = ‘TAGGAG’) OR (SS.pattern = ‘AAGGAG’) OR (SS.pattern = ‘GAGGAG’)) AND G.species = ‘Homo sapiens’. Query for the number of genes having a NAGGAG motif with the GAG within the coding exon:

SELECT count(distinct GeneID) FROM Gene G, Transcript T, SS2Transcript2SED SS2Tr2SED, SpliceSite SS, SpliceEventData SED WHERE G.ID = T.GeneID AND T.ID = SS2Tr2SED.TranscriptID AND SS2Tr2SED.SpliceSiteID = SS.ID AND SS2Tr2SED.SpliceEventDataID = SED.ID AND SS.Type = ‘acceptor’ AND SED.NumEESTs> = 1 AND SED.NumIESTs = 0 AND ((SS.pattern = ‘CAGGAG’) OR (SS.pattern = ‘TAGGAG’) OR (SS.pattern = ‘AAGGAG’) OR (SS.pattern = ‘GAGGAG’)) AND G.species = ‘Homo sapiens’;

Query for the number of genes having a NAGGAG motif with the GAG within the coding exon and with the intron phase = 0:

SELECT count(distinct GeneID) FROM Gene G, Transcript T, SS2Transcript2SED SS2Tr2SED, SpliceSite SS, SpliceEventData SED WHERE G.ID = T.GeneID AND T.ID = SS2Tr2SED.TranscriptID AND SS2Tr2SED.SpliceSiteID = SS.ID AND SS2Tr2SED.SpliceEventDataID = SED.ID AND SS.Type = ‘acceptor’ AND SED.NumEESTs> = 1 AND SED.NumIESTs = 0 AND ((SS.pattern = ‘CAGGAG’) OR (SS.pattern = ‘TAGGAG’) OR (SS.pattern = ‘AAGGAG’) OR (SS.pattern = ‘GAGGAG’)) AND SED.phaseUTR like ‘intron phase 0’ AND G.species = ‘Homo sapiens’.

## Supporting Information

Figure S1 Characterization of the glycosylation pattern of CFTR-del831-873 protein. Western blot analysis of HEK293 cells transiently transfected with CFTR-WT, CFTR-del831-873, or with the empty vector (mock). Lysates were incubated in the presence (+) or absence (−) of N-Glycosidase F. Filled, empty and shaded arrowheads indicate the fully-glycosylated (C band), core-glycosylated (B band) and non-glycosylated (A band) CFTR, respectively. Grey arrowhead indicates mutant protein.
(0.33 MB TIF)
