# Supplementary figures and images for: Alternative Splicing at a NAGNAG Acceptor Site as a Novel Phenotype Modifier
Source: PLoS Genet. 2010 Oct 7;6(10):e1001153. doi: 10.1371/journal.pgen.1001153 (PMC2951375; doi:10.1371/journal.pgen.1001153)

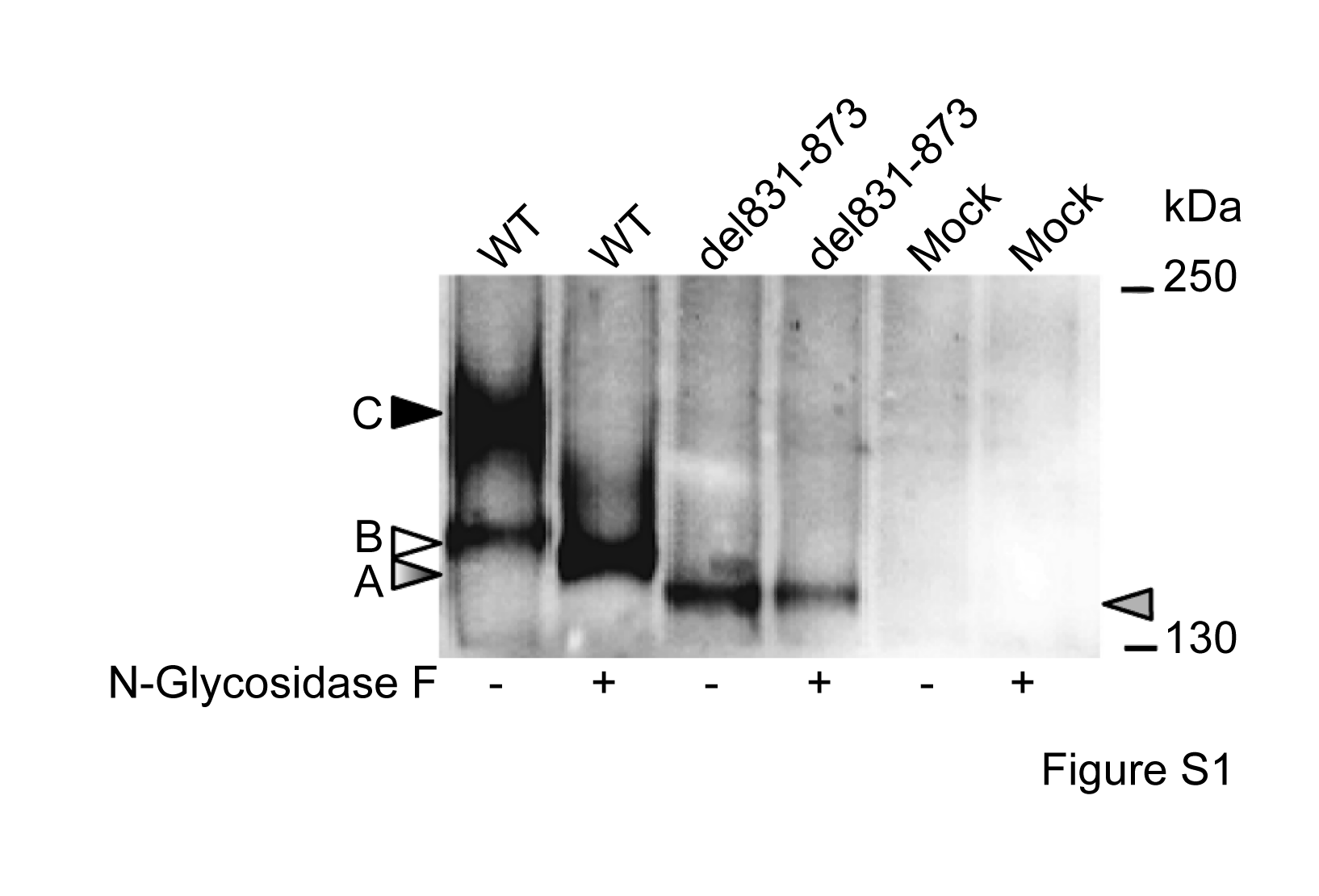

Supplement: Figure S1 — Characterization of the glycosylation pattern of CFTR-del831-873 protein. Western blot analysis of HEK293 cells transiently transfected with CFTR-WT, CFTR-del831-873, or with the empty vector (mock). Lysates were incubated in the presence (+) or absence (−) of N-Glycosidase F. Filled, empty and shaded arrowheads indicate the fully-glycosylated (C band), core-glycosylated (B band) and non-glycosylated (A band) CFTR, respectively. Grey arrowhead indicates mutant protein. (0.33 MB TIF) [file pgen.1001153.s001.tif]
